# Alcoholic cirrhosis in Denmark – population-based incidence, prevalence, and hospitalization rates between 1988 and 2005: A descriptive cohort study

**DOI:** 10.1186/1471-230X-8-3

**Published:** 2008-02-09

**Authors:** Peter Jepsen, Hendrik Vilstrup, Henrik T Sørensen

**Affiliations:** 1 Department of Clinical Epidemiology, Aarhus University Hospital, Aarhus, Denmark; 2 Department of Medicine V (Hepatology and Gastroenterology), Aarhus University Hospital, Aarhus, Denmark

## Abstract

**Background:**

Denmark has one of the highest alcohol consumption rates in Northern Europe. The overall per capita alcohol consumption has been stable in recent decades, but surveys have indicated that consumption has decreased in the young and increased in the old. However, there is no recent information on the epidemiology of alcoholic cirrhosis. We examined time trends in incidence, prevalence, and hospitalization rates of alcoholic cirrhosis in Denmark between 1988 and 2005.

**Methods:**

We used data from a nationwide population-based hospital registry to identify all Danish citizens with a hospital diagnosis of alcoholic cirrhosis. We computed standardized incidence rates, prevalence and hospitalization rates of alcoholic cirrhosis within the Danish population. We also computed the number of hospitalizations per alcoholic cirrhosis patient per year.

**Results:**

From 1988 to 1993, incidence rates for men and women of any age showed no clear trend, and after a 32 percent increase in 1994, rates were stable throughout 2005. In 2001–2005, the incidence rates were 265 and 118 per 1,000,000 per year for men and women, respectively, and the prevalence rates were 1,326 and 701 per 1,000,000. From 1994, incidence, prevalence, and hospitalization rates decreased for men and women younger than 45 years and increased in the older population, although the latter finding might be partly explained by changes in coding practice. Men and women born around 1960 or later had progressively lower age-specific alcoholic cirrhosis incidence rates than the generations before them. From 1996 to 2005, the number of hospitalizations per alcoholic cirrhosis patient per year increased from 1.3 to 1.5 for men and from 1.1 to 1.2 for women.

**Conclusion:**

From 1988 to 2005, alcoholic cirrhosis put an increasing burden on the Danish healthcare system. However, the decreasing incidence rate in the population younger than 45 years from 1994 indicated that men and women born around 1960 or later had progressively lower incidence rates than the generations before them. Therefore, we expect the overall incidence and prevalence rates of alcoholic cirrhosis to decrease in the future.

## Background

Alcohol consumption is the main determinant for liver cirrhosis occurrence in Western countries, yet population-based studies of the incidence of alcoholic liver disease are scarce [1]. Denmark has one of the highest alcohol consumption rates in Northern Europe [2], and there is ecological evidence that cirrhosis mortality rates follow alcohol consumption rates [2-4]. The Danish per adult (>14 years) alcohol consumption increased markedly from 1965 to 1975, but it has been nearly constant since then [5]. However, such overall rates may conceal changes within the population, and surveys conducted over the recent decades have indicated that alcohol consumption decreased in the young, but increased in the old [6-8]. Furthermore, even if cirrhosis mortality rates do follow alcohol consumption rates, cirrhosis incidence rates may not do so because the prognosis for cirrhosis patients is improving [9,10]. Thus, there is considerable uncertainty about the incidence of alcoholic cirrhosis in Denmark after 1985 [11], and, as a consequence, about the prevalence and hospitalization rates, too. We used the Danish nationwide population-based hospital registries to examine time trends in these rates [12,13].

## Methods

### Data sources

Denmark's population of 5.3 million persons receives free tax-supported healthcare. The National Patient Registry was established in 1977 and stores data from hospital contacts. Data from all inpatient admissions to non-psychiatric hospitals in Denmark have been recorded in the registry since 1977. In 1987, data from outpatient visits were added to the registry, but registration was not complete before 1995. From 1995, data from emergency room visits were added. The data from each hospital contact includes the type of contact (inpatient, outpatient, or emergency room), the patient's personal identification number, gender, age, municipality of residence, dates of admission and discharge (outpatients: first and last visit), and up to 20 diagnoses, one of which is designated the primary diagnosis, coded according to ICD8 (International Classification of Diseases, 8^th ^revision) until 1993 and ICD10 from 1994 throughout 2005. The hospital diagnoses are given at  discharge by the physician who discharges the patient [13]. Denmark's Civil Registration System updates the vital status of all Danish citizens on a daily basis, including date of death or emigration. Individual-level data from the National Patient Registry and Civil Registration System can be linked through the unique personal identification number.

Data from all pathologic examinations conducted in Denmark since 1997 are recorded in the Pathology Registry. The information is supplied by departments of pathology and includes biopsy date, SNOMED (Systematized Nomenclature of Medicine) codes for specimen type and pathology diagnoses, and a description of the pathologist's findings. All demographic information used in our study was obtained from the government office Statistics Denmark [14].

### Alcoholic cirrhosis patients

We identified all patients with a first-time diagnosis of alcoholic cirrhosis (ICD8: 571.09; ICD10: K70.3) from any type of hospital contact between 1 January 1977 and 31 December 2005. Patients were followed from the admission date of their earliest such hospital contact until death or censoring upon emigration or on 31 December 2005.

### Statistical analysis

#### Incidence

The incidence rate describes the rate at which new cases of alcoholic cirrhosis are diagnosed [15]. It was defined and computed as the number of patients with a first-time hospital diagnosis of alcoholic cirrhosis in a particular year divided by the total number of Danish citizens at the beginning of that year. The incidence rate for a range of years was obtained by summing the numerator and denominator for each year. Gender- and age-specific (younger than 45 years, 45–64 years, 65 years or older) incidence rates were computed by restricting both numerator and denominator accordingly. Likewise, we computed incidence rates within each of three geographical areas (Copenhagen [municipality codes 101 and 147], Jutland [municipality codes 501 through 861], and the remainder of Denmark). All incidence rates were age-standardized to the Scandinavian standard population [16], and 95% confidence intervals were based on Poisson distributions.

To explore whether the gradual inclusion of data from outpatient and emergency room visits to the National Patient Registry affected incidence rates, we computed incidence rates using only data from inpatient admissions. To explore whether any changes in the practice of coding comorbidities affected our findings, we computed incidence rates defining the study population as patients with alcoholic cirrhosis as a primary diagnosis.

#### Prevalence

The prevalence rate describes the proportion of the Danish population diagnosed with alcoholic cirrhosis [15]. It was defined and computed as the number of patients in the alcoholic cirrhosis cohort at the end of a particular year divided by the number of Danish citizens at the beginning of that year. Gender- and age-specific prevalence rates were computed as described above.

#### Hospitalization

The hospitalization rate describes the total number of inpatient admissions for patients diagnosed with alcoholic cirrhosis. It was defined and computed as the total number of inpatient admissions for the members of the alcoholic cirrhosis cohort in a particular year divided by the number of Danish citizens at the beginning of that year. Gender- and age-specific hospitalization rates were computed as described above. Transfers between hospital departments were not counted as new admissions. Dividing the hospitalization rate by the prevalence rate yielded the annual number of inpatient admissions per alcoholic cirrhosis patient.

#### Minimizing bias in presented rates

We underestimated the prevalence rates because some patients were diagnosed with alcoholic cirrhosis before the National Patient Registry was established in 1977. Also, we overestimated the incidence rates because some of these patients were given a hospital diagnosis of alcoholic cirrhosis after 1977. Both biases were largest in 1977 and then diminished. In a still unpublished study, based on these data, we found that the bias in incidence rates was less than 1 percent from 1988, and the bias in prevalence rates was less than 10 percent from 1996. Therefore, we presented incidence rates from 1988 and prevalence and hospitalization rates from 1996.

#### Validity of cirrhosis diagnoses

The positive predictive value of a hospital diagnosis of alcoholic cirrhosis was computed as the number of patients with a liver biopsy (SNOMED code T56xxx) containing incipient or manifest liver cirrhosis (determined from the pathologist's description or from SNOMED codes [M495xx except M49590 and M49591, or M496xx except M49620]) divided by the number of patients with a first-time hospital diagnosis of alcoholic cirrhosis between 1 January 1997 and 31 December 2005 and a liver biopsy obtained during that hospital contact. The 95% confidence interval for the positive predictive value was based on the binomial distribution.

## Results

### Incidence

A total of 16,745 patients were diagnosed with alcoholic cirrhosis between 1988 and 2005. The incidence rate of alcoholic cirrhosis in that period was more than twice as high for men as for women (Table 1). From 1988 through 1993, rates for men and women of any age showed no clear trend, but the 1994 rates for men and women were 32 percent higher than the 1993 rates (from 204.9 to 270.3 for men; from 88.9 to 117.5 for women). This marked increase was followed by stable incidence rates throughout the 1994–2005 period for both genders (Table 1). The incidence rate peaked at 50–55 years of age for both genders, but between 1988 and 2005 the median age at diagnosis rose from 53 to 56 years.

**Table 1:** Standardized incidence, prevalence, and hospitalization rates of alcoholic cirrhosis in Denmark.

			**1988–1993**	**1994–1995**	**1996–2000**	**2001–2005**
< 45 years	Men	Incidence rate	66 (62–71)	84 (74–93)	67 (62–73)	49 (45–54)
		Prevalence rate	-	-	253 (243–264)	170 (162–179)
		Hospitalization rate	-	-	410 (397–423)	302 (291–313)
	Women	Incidence rate	30 (27–34)	40 (33–47)	36 (32–40)	26 (22–29)
		Prevalence rate	-	-	129 (121–136)	95 (88–101)
		Hospitalization rate	-	-	201 (192–211)	185 (176–194)
45–64 years	Men	Incidence rate	469 (446–492)	702 (655–749)	691 (662–719)	717 (689–745)
		Prevalence rate	-	-	3440 (3377–3504)	3620 (3558–3682)
		Hospitalization rate	-	-	4350 (4279–4421)	5124 (5050–5199)
	Women	Incidence rate	214 (199–230)	298 (267–328)	296 (277–314)	331 (312–350)
		Prevalence rate	-	-	1730 (1685–1775)	1887 (1841–1932)
		Hospitalization rate	-	-	1878 (1831–1925)	2260 (2211–2310)
≥ 65 years	Men	Incidence rate	268 (245–291)	383 (335–432)	398 (367–430)	494 (460–529)
		Prevalence rate	-	-	2480 (2401–2558)	2837 (2755–2920)
		Hospitalization rate	-	-	2960 (2874–3046)	3642 (3548–3735)
	Women	Incidence rate	99 (87–112)	121 (96–145)	151 (133–169)	171 (152–190)
		Prevalence rate	-	-	1280 (1229–1332)	1537 (1481–1593)
		Hospitalization rate	-	-	1207 (1157–1257)	1452 (1397–1506)

Total	Men	Incidence rate	189 (182–196)	271 (257–286)	260 (251–268)	265 (257–274)
		Prevalence rate	-	-	1295 (1276–1314)	1326 (1307–1345)
		Hospitalization rate	-	-	1675 (1654–1697)	1875 (1853–1897)
	Women	Incidence rate	84 (79–89)	113 (104–122)	114 (108–119)	118 (112–124)
		Prevalence rate	-	-	656 (642–669)	701 (688–715)
		Hospitalization rate	-	-	731 (717–745)	843 (828–858)

Incidence and hospitalization rates are per 1,000,000 population per year. Prevalence rates are per 1,000,000 population. Parentheses show the 95% confidence interval.

**Figure 1:**
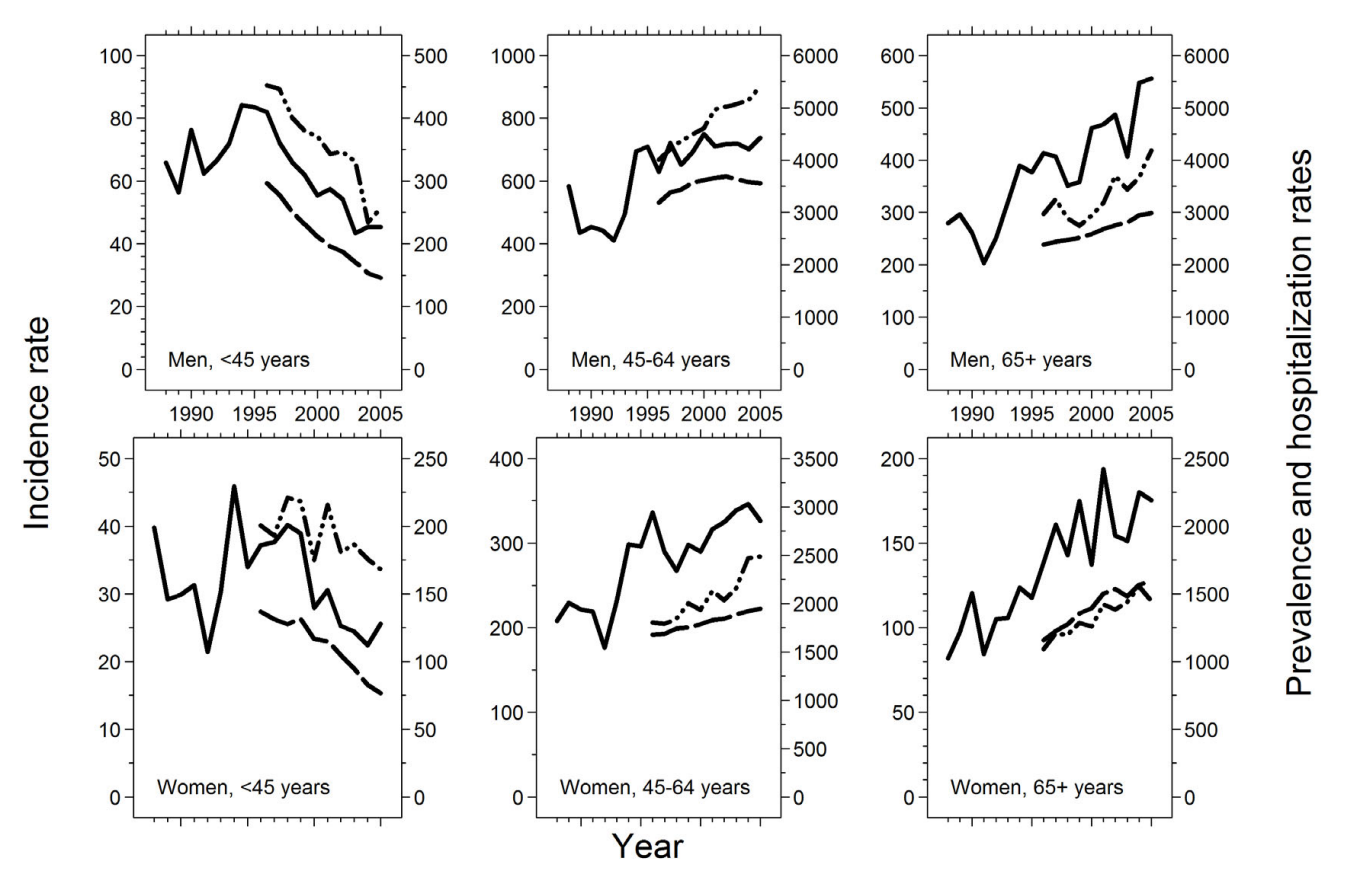
**Incidence rates (solid lines), prevalence rates (dashed lines), and inpatient hospitalization rates (dash-dot-dot lines) of alcoholic cirrhosis by gender and age**. All rates are standardized to the Scandinavian standard population. Incidence and hospitalization rates are per 1,000,000 population per year, prevalence rates are per 1,000,000 population. The Danish per adult (>14 years) alcohol consumption increased from 7 to 12 liters from 1965 to 1975, was 12 liters from 1975 to 2001, and 11 liters thereafter.

Among men and women younger than 45 years, the incidence rate peaked in 1994 and then decreased. Among men and women aged 45–64 years, the incidence rate increased sharply from 1992 to 1994, after which time it increased only slightly. Among men and women aged 65 years or older, the incidence rates increased throughout the 1988 to 2005 period (Table 1 and Figure 1).

Men and women born in 1957–1965, who were aged 40–44 years in 2001–2005, had a consistently lower age-specific incidence rate of alcoholic cirrhosis than earlier birth cohorts. Continuing this trend, subsequent birth cohorts had progressively lower age-specific incidence rates (Table 2). Furthermore, men and women who were born in 1917–25, i.e. were aged 65–69 years in 1986–90, and earlier birth cohorts had lower age-specific incidence rates than later birth cohorts (Table 2).

**Table 2:** Incidence rates of alcoholic cirrhosis, per 1,000,000 population, for men (top) and women (bottom) by age and calendar year.

Men	20–24	25–29	30–34	35–39	40–44	45–49	50–54	55–59	60–64	65–69	70–74	75–90	80+
1986*–1990	0	**18**	79	203	304	432	493	561	483	389	289	226	150
1991–1995	0	15	**74**	230	352	445	583	629	575	464	316	182	131
1996–2000	0	12	47	**162**	392	536	664	805	807	590	453	245	116
2001–2005	1	5	32	123	**287**	592	726	776	810	741	535	323	173

Women	20–24	25–29	30–34	35–39	40–44	45–49	50–54	55–59	60–64	65–69	70–74	75–90	80+

1986*–1990	0	**11**	44	102	142	191	210	265	221	165	83	77	24
1991–1995	1	4	**42**	101	147	230	255	271	221	162	96	85	59
1996–2000	2	5	22	**98**	201	251	302	327	312	236	173	72	50
2001–2005	3	1	10	49	**171**	274	314	384	370	277	163	109	57

* Data for 1986 and 1987 were not used.

Age- and calendar year-intervals are chosen so that a birth cohort can be followed through time by going diagonally down and to the right. For example, those who were aged 25–29 years in 1986–1990 were born between 1957 and 1965, with the majority born in 1961. The incidence rates in bold letters are all for that birth cohort.

The steep increase in alcoholic cirrhosis incidence from 1993 to 1994 for both genders was seen in all three geographic areas. Restricting our data to inpatient hospitalizations lowered the incidence rates for 1994 and all following years by approximately 10 percent, but did not affect time trends. Restricting our study population to patients with alcoholic cirrhosis as a primary diagnosis eliminated the slightly increasing trends from 1994 among men and women aged 45–64 years and attenuated the increasing trends among men and women aged 65 years or older.

### Prevalence

The prevalence rates for men and women of any age increased slightly from 1996 to 2005 (Table 1). The gender- and age-specific prevalence rates followed the time trends in incidence rates (Table 1 and Figure 1).

### Hospitalization

The hospitalization rates for men and women of any age increased from 1996 to 2005 (Table 1), and the number of inpatient hospitalizations per alcoholic cirrhosis patient per year increased from 1.3 to 1.5 for men and from 1.1 to 1.2 for women. Time trends in gender- and age-specific hospitalization rates followed the time trends in prevalence rates, but the number of inpatient hospitalizations per alcoholic cirrhosis patient increased in all gender- and age-categories, except among women aged 65 years or older (Figure 1). It was highest for men and women younger than 45 years, who had 2.2 and 1.8 inpatient hospitalizations per alcoholic cirrhosis patient in 2005, respectively.

### Validity of cirrhosis diagnoses

We reviewed information from 516 liver biopsies, and 401 contained incipient or manifest cirrhosis yielding a positive predictive value of 78% (95% confidence interval = 74% to 81%). The positive predictive value was constant during the 1997–2005 period: 78% in 1997–1999, 77% in 2000–2002, and 79% in 2003–2005.

## Discussion

In this nationwide population-based study of the 1988–2005 period we found that, in the population younger than 45 years, the incidence rate of alcoholic cirrhosis peaked in 1994 and then decreased. It appeared that men and women born in 1960 or later had progressively lower age-specific incidence rates than earlier birth cohorts. In the population aged 45–64 years, a steep increase in incidence rate from 1993 to 1994 was followed by a less markedly increasing trend. In the population aged 65 years or older, the incidence rate increased throughout the 1988–2005 period. From 1996 onwards, the trends in prevalence and hospitalization rates generally followed the incidence trends, but the number of inpatient hospitalizations per alcoholic cirrhosis patient per year increased.

The primary strength of our study is that it was based on nearly three decades of individual-level data from a nationwide population-based hospital registry with complete follow-up [12].

We may have underestimated the incidence rate because we failed to include alcoholic cirrhosis patients who were never hospitalized. However, we assume that this bias is small because Danish clinical practice is to refer all patients with signs or symptoms of cirrhosis to diagnostic workup and treatment in a public hospital. Another limitation is that hospital diagnoses may be wrong, so that patients with cirrhosis are not registered with the diagnosis (low completeness of registration), or patients registered with the diagnosis do not have cirrhosis (low positive predictive value of registration). A study of patients referred to Danish non-specialized medical departments in 1985–1989 found a completeness of 93 percent and a positive predictive value of 85 percent for a hospital diagnosis of cirrhosis, using a gold standard of either liver biopsy or a combination of clinical findings [17]. The different gold standards are likely to explain the lower positive predictive value in our study [18]. Therefore, the incidence rates may be slightly inaccurate, but the time trends in them were probably not substantially biased.

The prevalence rates depended on accurate incidence rates as well as on accurate information on the survival time of alcoholic cirrhosis patients, which was ensured by the Civil Registration System [12]. They could be underestimated by as much as 10 percent in 1996, but by gradually smaller amounts thereafter. Thus, the prevalence rate increases seen among men and women older than 45 years could be due to bias, but the decreasing trend in prevalence rates among younger men and women could not. The hospitalization rates depended on accurate prevalence rates and emphasize that cirrhosis is a risk factor for a number of conditions that require hospitalization [19-23]. The increasing trend in the number of hospitalizations per alcoholic cirrhosis patient is probably unbiased and clearly indicates that alcoholic cirrhosis put an increasing burden on the Danish tax-funded healthcare system.

The 32 percent increase in alcoholic cirrhosis incidence from 1993 to 1994 is striking. The addition of data from outpatient and emergency room visits to the National Patient Registry increased the incidence rates by around 10 percent, accounting for a third of the increase. We also found that the increase was seen throughout Denmark, and did not depend on whether we considered only primary diagnoses. Increasing diagnostic activity and sensitivity could have contributed, but they could not explain such an abrupt increase, and they are not consistent with the increasing age at diagnosis during the study period. We find it likely that the shift from ICD8 to ICD10, which took place on 1 January 1994, may somehow have contributed although both versions contain one unambiguous code for alcoholic cirrhosis. Thus, part of the increase is an artifact caused by changes in the National Patient Registry, but our findings indicate that the incidence rate did in fact increase. The most likely reason is that the 1930–50 birth cohorts reached the typical age of alcoholic cirrhosis diagnosis, 45–64 years, around 1994. It is very unlikely that an increasing prevalence of hepatitis C infection, which is a risk factor for cirrhosis among alcohol abusers [24], could have contributed because hepatitis C infection has a very low prevalence in the Danish population, 0.2 percent in 1997 [25].

Changes in doctors' coding practice also affected our findings. In earlier years, alcoholic cirrhosis was less likely to be coded if it was not the primary diagnosis, as indicated by the attenuation of the increasing incidence trends when we considered only primary diagnoses. Thus, we may have underestimated the incidence rates in the beginning of the study period, but such a bias could not explain the decreasing trend among men and women younger than 45 years.

Our findings are consistent with data from Statistics Denmark and findings from surveys. In Denmark, the per adult (>14 years) alcohol consumption increased from 7 liters in 1965 to 12 liters in 1975 [5], and it is likely that those born between 1925 and, say, 1950, were responsible for this increase. The per adult consumption was stable from 1975 to 2005, and this may be explained by a continued high consumption by the 1925–1950 birth cohort combined with a low consumption by those born after 1960. Based on three surveys of Danish men and women aged 30, 40, 50, or 60 years in 1982–84, 1986–87, and 1991–92, Gerdes et al. found that alcohol consumption decreased among 30-year-olds, particularly from the 1956–57 birth cohort to the 1961–62 birth cohort [6]. Såbye-Hansen et al. found that men and women aged 15 to 39 years consumed less alcohol in 1992 than in 1979, whereas men and women aged 40 to 79 years consumed more [7]. Similarly, Bjørk et al. surveyed men and women in 1987, 1994, 2000, and 2003 [8] and found an increase in alcohol consumption among men and women aged 50 years or older and a decrease among men and women aged 16–49 years. Thus, we find it plausible that Danes born around 1960 or later consume less alcohol than the generations before them. The relatively high alcohol consumption by the generations born before 1960 may be explained by social and cultural changes during their lifetime, particularly for women [7,8].

## Conclusion

We found that, from 1988 to 2005, the increasing prevalence of alcoholic cirrhosis and the increasing number of inpatient hospitalizations per alcoholic cirrhosis patient put an increasing burden on the Danish healthcare system. However, we found a decreasing incidence rate in the population younger than 45 years from 1994, and this indicated that men and women born around 1960 or later had progressively lower incidence rates than the generations before them. We forecast that, as these men and women grow older, the decreasing incidence trend will spread to older age groups, and we will see a decrease in the overall incidence rate of alcoholic cirrhosis when they reach the typical age of alcoholic cirrhosis onset, 45–64 years. This will eventually be followed by a decreasing prevalence of alcoholic cirrhosis in the Danish population. These findings are important for public health and healthcare planning.

## Competing interests

The author(s) declare that they have no competing interests.

## Authors' contributions

All authors conceived and designed the study. PJ analyzed the data. All authors interpreted the data. PJ drafted the manuscript, and HV and HTS revised it. All authors have seen and approved the final manuscript.

## Pre-publication history

The pre-publication history for this paper can be accessed here:


